# Response of seed germination and seedling emergence of *Haloxylon ammodendron* to rain frequency and temperature change from four desert ecosystems, Northwest China

**DOI:** 10.1093/aobpla/plac048

**Published:** 2022-12-27

**Authors:** Yajuan Zhu, Zhiqing Jia, Guoje Wang, Husen Ning, Xiaomin Ji, Qinghong Luo

**Affiliations:** Research Center of Desertification, Institute of Ecological Conservation and Restoration, Chinese Academy of Forestry, Beijing 100091, China; Research Institute of Forestry, Chinese Academy of Forestry, Beijing 100091, China; Department of Plant Science, The Pennsylvania State University, University Park, PA 16802, USA; Institute of Forestation and Sand Control, Xinjiang Academy of Forestry, Urumqi 830063, China; Institute of Forestation and Sand Control, Xinjiang Academy of Forestry, Urumqi 830063, China; Institute of Forestation and Sand Control, Xinjiang Academy of Forestry, Urumqi 830063, China

**Keywords:** Alternating temperature, constant temperature, rain frequency, seed germination, seedling emergence

## Abstract

Climate change will result in variation of rain frequency and amount and warming in arid zones, which is expected to affect seed germination and seedling emergence in desert ecosystems. However, the effects of unpredictable rainfall and increasing temperature on seed germination and seedling emergence of dominant desert plants remain unclear across different deserts, which are important for population regeneration and community succession in desert ecosystems. Seed germination and seedling emergence of *Haloxylon ammodendron* across four deserts in Northwest China were examined at different rain frequencies with same total amount, and constant and alternating temperatures, to investigate their response to climate change. Rain frequency determined seed germination and seedling emergence of *H. ammodendron* in the Tengger Desert, Badain Jaran Desert, Gurbantonggut Desert and Mutthar Desert, which was maximal at rain frequency of 10 times per month and decreased with a decrease of rain frequency. Temperature was not a restricting factor for seed germination of *H. ammodendron* in the Tengger Desert, Badain Jaran Desert and Gurbantonggut Desert, varying from 10 °C to 25 °C and from 20/10 °C to 30/15 °C, respectively. However, the highest temperature of 25 °C and 30/15 °C inhibited seed germination of *H. ammodendron* in the Mutthar Desert. Thus, *H. ammodendron* has an opportunistic germination strategy. Under climate change in the future, seed germination and seedling emergence of *H. ammodendron* would be restricted by the combination of less frequent rainfall and increased temperature in desert ecosystems. The regeneration of the *H. ammodendron* community should be promoted by irrigation and seedling transplant.

## Teaser

In the deserts of Central Asia and Northwest China, *Haloxylon ammodendron*(saxual) is a dominant shrub. We collected seed from four deserts in Northwest China and tested their germination in a greenhouse and incubator. We found that these seeds germinated better when they were irrigated once every 3 days with 50 mm monthly rain. They germinated well in temperatures similar to the spring and early summer, but would be inhibited by higher temperatures in the summer (30 °C or 30/15 °C). Therefore, its regeneration would be restricted by lower rain frequency and higher temperatures under future climate change.

## Introduction

Global climate change due to the greenhouse effect of elevated CO_2_ includes changing precipitation regimes and warming (IPCC 2013). A desert ecosystem is highly sensitive to climate change because plant species are already struggling in an extreme environment, including drought and high temperature. Since water and temperature are two key factors for plant survival and growth in a desert ecosystem, the changes of hydrothermal availability are expected to have an influence on the structure and function of the desert plant community (Liu *et al.* 2016; Bai *et al.* 2020; Wang *et al.* 2018a).

Seed germination is the key stage in the establishment of plant species, which is also the most sensitive stage to environmental change in the life cycle (Baskin and Baskin 2014). Plant species have different seed germination strategies to ensure their survival in the desert ecosystem (Gutterman 2002). Some desert plants have an opportunistic strategy and produce seeds that germinate soon after rain in the growing season (Tobe *et al.* 2005b). However, other desert plants, especially perennial herbs and shrubs, have a cautious strategy (Commander *et al.* 2017) and produce few seeds that germinate only in specific conditions. Seed germination of desert plants often is restricted by many environmental factors, such as moisture, temperature, light and sand burial (Li *et al.* 2012; Zhu *et al.* 2014), depending on the species. Germination time may be limited to spring or autumn, or seeds may germinate throughout the growing season (Baskin and Baskin 2014). Therefore, investigating seed germination of desert plants will increase our understanding of their adaptive strategy to arid climate and result in sustainable management practices of desert vegetation.

In desert ecosystems, precipitation is usually low and unpredictable in its spatiotemporal pattern (Gutterman 2002), and mainly occurs as sporadic rainfall during the growing season, restricting seed germination and seedling emergence (Gillespie *et al.* 2004; Tobe *et al.* 2006). It has been predicted that under global warming, less frequent, but higher precipitation events will increase in the future (Allan and Soden 2008; IPCC 2013). The frequency, intensity and spatial pattern of rain have important effects on seed germination and seedling establishment in the desert (Gutterman 2002). Seeds of desert shrubs may need continuous rain events or heavy rainfall to trigger seed germination (Schwinning and Sala 2004). For example, high seedling emergence of two *Artemisia* semi-shrubs, *Caragana korshinskii* and *Hedysarum fruticosum* occurred at 10 mm, 5 and 7.5 mm and 5 mm rain every 3 days in Mu Us Sandy Land (Zheng *et al.* 2004, 2005). Seedling emergence of three *Artemisia* semi-shrubs was maximal with an initial 16 mm and subsequent 3 mm of irrigation at 1-day intervals in deserts of Northwest China (Tobe *et al.* 2006). Watering level for maximal seedling emergence of *C. korshinskii*, *Hedysarum leave* and *Artemisia ordosica* in Mu Us Sandy Land was 10 mm, 10–20 mm and 15–20 mm, respectively (Zheng *et al.* 2009). Moreover, with increased rain amount, seed germination and seedling emergence generally increased, such as three *Artemisia* semi-shrubs (Tobe *et al.* 2006) and *Artemisia sphaerocephala* in Mu Us Sandy Land (Yang *et al.* 2012), and *Reaumuria soongarica* in Badain Jaran Desert (Shan *et al.* 2018). Rain frequency also influenced seedling emergence. With a decrease in rain frequency, seedling emergence decreased, e.g. *A. ordosica* (Tobe *et al.* 2006). However, most studies about seed germination and seedling emergence in different rain patterns were performed for one species or for interspecific variation in one area. For widely distributed species across different areas, we still do not know the intraspecific variation of seed germination under different rainfall regime.

Temperature is another environmental factor that determines seed germination (Dürr *et al.* 2015). Moreover, the seasonal change of temperature primarily regulates germination in the field. Seeds of most shrubs in temperate desert ecosystems generally germinate over a wide range of temperatures, varying from 3 °C to 30/15 °C (Baskin and Baskin 2014). Seeds of some desert shrubs germinated well when they were incubated at constant temperature. For example, most seeds of two *Haloxylon* shrubs in China germinated at 5 °C to 30 °C (Tobe *et al.* 2000). Maximal seed germination of *Nitraria tangutorum* and *Nitraria spaerocarpa* occurred at 15 °C to 25 °C and at 20 °C and 25 °C, respectively (Wang and Zhang 2009). In Horqin Sandy Land, seeds of two *Artemisia* semi-shrubs germinated at 10 °C to 34 °C (Li *et al.* 2012); however, seeds of *Caragana micophylla* germinated well at 15 °C to 30 °C (Lai *et al.* 2019). Seeds of other desert shrubs germinated well at alternating temperatures. For example, seeds of *C. korshinskii* from Ordos Plateau germinated at 5/15 °C to 25/35 °C (Lai *et al.* 2015). Seeds of *Artemisia halodendron*, *C. korshinskii* and *C. microphylla* from the Horqin Sandy Land germinated well at 20/10 °C to 30/20 °C (Lai *et al.* 2016). Seed germination of 11 species from a desert steppe in Inner Mongolia increased as temperature was increased from 0/12 °C to 15/27 °C, whereas another nine species germinated well at the lowest temperature of 0/12 °C (Yi *et al.* 2019). Moreover, alternating temperatures were more favourable for germination than constant temperature of some psammophytes, such as *C. korshinskii* (Baskin and Baskin 2014). However, most previous studies about seed germination at different temperatures were carried out for one species or for the interspecific variation in one area. For widely distributed species, there were few studies about the intraspecific variation of seed germination from different areas, e.g. the differentiation in seedling emergence of *Coleogyne ramosissima* in Mojave Desert and Colorado Plateau (Meyer and Pendleton 2005).

*Haloxylon ammodendron* is a dominant shrub or small tree in the deserts of Northwest China and central Asia, which is widely distributed in sandy desert, Gobi, clay desert and salt desert (Ma 1991). This species plays an important role in the ecological function of the desert ecosystem, such as sand dune stabilization, carbon sequestration and biodiversity conservation (Gao *et al.* 2010; Zhu and Jia 2011). Moreover, it has important economic value as the host plant for *Cistanche deserticola*, a Chinese traditional herb (Ma 1991). The species has been widely used in land desertification control in Northwest China and central Asia. Regeneration of the *H. ammodendron* community relies on seed germination and seedling establishment. Previous studies showed that seed germination of *H. ammodendron* was affected by temperature, which was suitable at 10 °C and lowest at 30 °C; however, it was not affected by light or darkness (Huang *et al.* 2003). High seedling emergence of *H. ammodenron* resulted from 8 to 20 mm of irrigation (Tobe *et al.* 2005a). However, we still do not know the effect of rain frequency and alternating temperatures on seed germination of this dominant shrub in the desert ecosystem. To obtain a better understanding of the adaptive strategy of dominant desert shrubs to a harsh environment, we investigated the effects of rain frequency and temperature change on seed germination of *H. ammodendron* and discussed the possible impact of climate change on its population regeneration. Our research will give theoretical support to the sustainable management of desert vegetation under climate change.

## Materials and Methods

### Seed collection

In late October and early November of 2019, seeds (utricles) of *H. ammodendron* were collected in four desert ecosystems from east to west in China, e.g. Tengger Desert, Badain Jaran Desert, Gurbantonggut Desert and Mutthar Desert, which are the main distribution areas for *H. ammodendron* in the arid zone of Northwest China. The location and climate of the four seed sources are shown in Table 1. The four deserts have a typical temperate continental climate. Tengger Desert and Badain Jaran Desert are located on Alxa Plateau of Mongolian Plateau, which has less mean annual precipitation than the other two deserts. Gurbantonggut Desert and Mutthar Desert are located in Junggar Basin of central Asia. The mean daily air temperature is highest in July in the Mutthar Desert. Seeds were manually shaken from shoots of *H. ammodendron*, and then stored in a cotton bag at room temperatures varying from 13 °C to 22 °C for 5 months.

**Table 1.** The location, climate and seed mass in the four deserts where seed of *Haloxylon ammodendron* were collected in Northwest China.

Seed source	Longitude, latitude	Elevation (m)	MAP (mm)	MAT (°C)	*T*_max_ (°C)	*T*_min_ (°C)	Seed mass (g)
Tengger Desert	39°34.13ʹN, 105°45.03ʹE	1048	115	8.3	23.5	−9.2	3.25 ± 0.05
Badain Jaran Desert	39°24.53ʹN, 102°46.02ʹE	1236	113	8.4	24.5	−7.8	2.65 ± 0.03
Gurbantonggut Desert	44°11.21ʹN, 89°32.03ʹE	651	150	5.5	22.6	−18.9	3.63 ± 0.05
Mutthar Desert	44°32.75ʹN, 83°23.41ʹE	366	167	7.8	26.3	−19.2	2.73 ± 0.07

MAP: mean annual precipitation; MAT: mean annual air temperature; *T*_max_: mean daily air temperature in July; *T*_min_: mean daily air temperature in January. Seed mass is the total mass of 1000 seeds.

Before this experiment, the pericarp and wing of *H. ammodendron* seeds were removed manually. Seed mass was measured by an electronic balance (0.01 g) for 1000 seeds with four replicates. The mean mass of 1000 *H. ammodendron* seeds from the Tengger Desert, Badain Jaran Desert, Gurbantonggut Desert and Mutthar Desert was 3.25, 2.65, 3.63 and 2.73 g, respectively (Table 1).

### Experimental design and measurement

Based on the field observation, most seeds of *H. ammodendron* germinated after rain from April to June. Thus, climate data of four seed sources were analysed to determine experiment conditions. On average, there were 2.00 to 3.71 times rainfall events in the four seed sources in April, 1.71 to 5.00 times in May, and 4.00 to 5.00 times in June (Table 2). Thus, four rain frequencies (10, 6, 3 or 2 times per month) were applied in our experiment, considering historical rainfall data and the decrease of rain frequency resulting from climate change in the future.

**Table 2.** Rain frequency (times per month) (mean ± SE, range) during the germination season in the four deserts in Northwest China.

Seed source	April	May	June	Mean
Tengger Desert	2.29 ± 1.25 (1‒4)	3.14 ± 1.35 (1‒5)	4.00 ± 1.63 (1‒6)	3.14 ± 1.53
Badain Jaran Desert	2.00 ± 1.53 (0‒4)	1.71 ± 0.95 (1‒3)	4.29 ± 1.98 (1‒6)	2.67 ± 1.88
Gurbantonggut Desert	3.71 ± 1.50 (2‒6)	4.29 ± 0.76 (3‒5)	4.86 ± 1.07 (4‒7)	4.29 ± 1.19
Mutthar Desert	3.71 ± 1.60 (1‒6)	5.00 ± 1.41 (2‒6)	5.00 ± 1.41 (4‒7)	4.57 ± 1.54

Seed germination tests were conducted from 12 April to 13 May in 2020 in a non-heated greenhouse at the Chinese Academy of Forestry. The substrate for seed germination was river sand passed through a sieve with the diameter of 2 mm. Plastic pots (15.6 cm diameter × 13.2 cm height) were filled with sand to within 1 cm of the top, and 25 seeds were planted uniformly with forceps at 1 cm depth in each pot because the highest seed germination percentage of *H. ammodendron* was obtained at 1 cm depth (Wang and Zhao 2015). There were four pots (replicates) for each treatment, totalling 64 pots in this experiment (4 rain frequencies × 4 seed sources × 4 replicates). A seedling was considered to be emerged when its first foliage leaf was 5 mm above sand surface. Daily air temperatures varied from 14 °C to 31 °C in the greenhouse during the experiment with the mean minimal value of 20.1 °C and the mean maximal value of 28.1 °C.

There were four watering frequencies (10, 6, 3 or 2 times per month) for *H. ammodendron* seeds from the four deserts with a monthly total rain of 50 mm, which was equivalent to 88, 147, 293 and 440 mL water each time, respectively. Seedling emergence was recorded daily for each pot, and the experiment was terminated after 30 days, at which time no seedlings had emerged for at least 5 consecutive days. All sand in each pot was passed through a sieve with 2 mm diameter to search for germinated but non-emerged seedlings, and then seed germination percentage was calculated for each treatment.

From April to June, maximum daily temperature in the four deserts varied from 17.62 °C to 21.01 °C, from 22.91 °C to 26.82 °C and from 26.90 °C to 32.44 °C; meanwhile, minimum daily temperature varied from 4.12 °C to 8.51 °C, from 9.33 °C to 14.11 °C and from 15.19 °C to 19.37 °C (Table 3). Thus, based on the air temperature data in the germination season, four constant temperatures (10 °C, 15 °C, 20 °C and 25 °C) and four alternating temperatures (20/10 °C, 25/10 °C, 25/15 °C and 30/15 °C) were used in our experiment.

**Table 3.** The air temperature (°C) during the germination season in the four deserts, Northwest China.

Seed source	*T*_max_ in April	*T*_min_ in April	*T*_max_ in May	*T*_min_ in May	*T*_max_ in June	*T*_min_ in June
Tengger Desert	17.62 ± 1.25	6.22 ± 0.25	22.91 ± 1.11	11.73 ± 1.02	26.90 ± 0.89	16.71 ± 0.84
Badain Jaran Desert	18.57 ± 0.93	5.61 ± 0.41	23.61 ± 1.22	11.03 ± 0.72	28.38 ± 1.17	16.69 ± 0.77
Gurbantonggut Desert	19.33 ± 1.39	4.12 ± 1.18	24.20 ± 1.24	9.33 ± 0.96	28.99 ± 1.11	15.19 ± 1.03
Mutthar Desert	21.01 ± 1.76	8.51 ± 1.66	26.82 ± 1.64	14.11 ± 1.36	32.44 ± 0.71	19.37 ± 0.83

*T*_max_: maximal daily temperature; *T*_min_: minimal daily temperature.

Seeds of *H. ammodendron* from the four deserts were tested for germination in the incubators in light (fluorescent tube with light intensity of 50 μmol m^−2^ s^−1^) at the Plant Ecophysiology Laboratory in Chinese Academy of Forestry. The test using four constant temperatures was conducted from 22 April to 7 May 2020; and the one using four alternating temperatures was conducted from 11 June to 26 June 2020. The period for high and low temperature was 12 h and 12 h in the alternating temperature treatments, respectively. Twenty-five seeds were uniformly placed on two layers of Whatman No. 1 filter paper and 2 mL distilled water in a 9 cm diameter plastic Petri dish using forceps. There were four Petri dishes (replicates) for each treatment and a total of 64 dishes in each experiments (4 temperatures × 4 seed sources × 4 replicates). A seed was considered to be germinated when the radicle appeared (1 mm) from the seed coat. Germination was examined daily, distilled water was added when necessary and the germinated seeds were removed from the Petri dish. The seed germination experiment was terminated after 15 days, at which time no seeds had germinated for at least 5 consecutive days.

### Statistical analysis

A completely randomized design was used in all experiments. Percentages of seed germination and seedling emergence were expressed as mean ± SE (standard error). The percentages were arcsine square root transformed before analysis, but untransformed data are shown in tables and figures. Two-way ANOVA at the 95 % probability level was conducted to compare the effects of rain frequency and seed source on the percentage of seed germination and seedling emergence of *H. ammodendron*, and the effects of constant temperature or alternating temperature and seed source on seed germination percentage (Sokal and Rohlf 1995). If ANOVA showed significant effects, Duncan’s test was used to determine the difference between treatments. All analysis were conducted by SPSS 19.0 for Windows (SPSS Inc., Chicago, IL, USA).

## Results

### Effects of rain frequency on seed germination and seedling emergence

**Figure 1.**
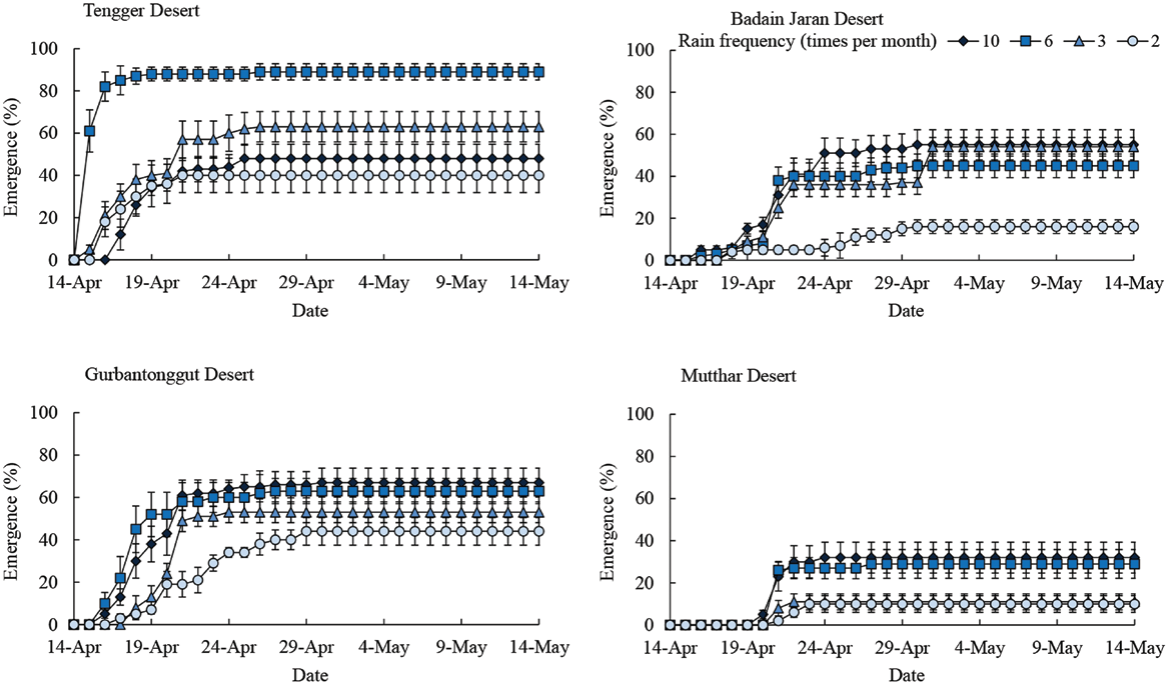
The cumulative daily seedling emergence of *Haloxylon ammodendron* seeds from four deserts at different rain frequencies.

Seedling emergence of *H. ammodendron* was delayed with decreasing rain frequency (Fig. 1). Tengger Desert seedlings of *H. ammodendron* emerged on the second day at the rain frequency of 6 and 3 times per month but on the fourth day at 2 times per month. Badain Jaran Desert seedlings of *H. ammodendron* emerged on the third day at the rain frequency of 10 and 6 times per month but on the fifth day at less frequent rain. Gurbantonggut Desert seedlings of *H. ammodendron* emerged on the third day at the rain frequency of 10 and 6 times per month but on the fourth day at less frequent rain. Mutthar Desert seedlings of *H. ammodendron* emerged on the seventh day at the rain frequency of 10 times per month but on the eighth day at less frequent rain. In general, seedling emergence of *H. ammodendron* was delayed for seeds from east to west, which was fastest for Tengger Desert seeds and slowest for Mutthar Desert seeds.

**Figure 2.**
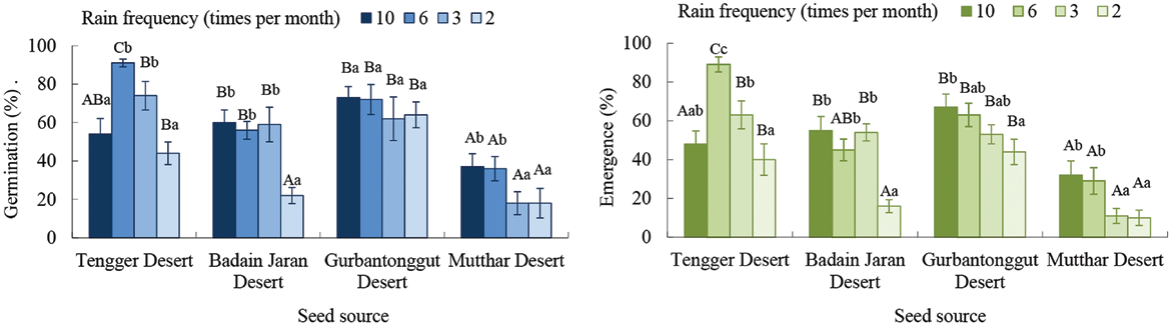
The final percentage of seed germination and seedling emergence of *Haloxylon ammodendron* from four deserts at different rain frequencies. Different upper-case letters indicate significant differences between seed sources and different lower-case letters indicate significant differences between rain frequencies.

Seed germination and seedling emergence percentage of *H. ammodendron* were significantly affected by rain frequency (*P* < 0.001), seed source (*P* < 0.001) and their interactions (*P* < 0.01, Table 4). Generally, seedling emergence declined with decreasing rain frequency (Fig. 1). The percentages of seed germination (91) and seedling emergence (89) of *H. ammodendron* seeds from the Tengger Desert were higher at the rain frequency of 6 times per month than those at other treatments. The percentages of seed germination and seedling emergence of *H. ammodendron* seeds from the Badain Jaran Desert were higher at the rain frequency of 10, 6 and 3 times per month than at 2 times per month. The percentages of seed germination (73 and 72) and seedling emergence (67 and 63) of *H. ammodendron* seeds from the Gurbantonggut Desert were higher at the rain frequency of 10 and 6 times per month than those at other treatments. The percentages of seed germination (37 and 36) and seedling emergence (32 and 29) of *H. ammodendron* seeds from the Mutthar Desert were higher at the rain frequency of 10 and 6 times per month than those at other treatments (Figs 1 and 2). At the rain frequency of 10 times per month, the percentages of seed germination and seedling emergence of *H. ammodendron* were higher in Badain Jaran Desert and Gurbantonggut Desert than the other two deserts. At the rain frequency of 6 times per month, the percentages of seed germination and seedling emergence were highest in Tengger Desert. At the rain frequency of 3 times per month, the percentages of seed germination and seedling emergence were higher in Tengger Desert, Badain Jaran Desert and Gurbantonggut Desert than in the Mutthar Desert. At the rain frequency of 2 times per month, the percentages of seed germination and seedling emergence were higher in Gurbantonggut Desert and Tengger Desert than in the other two deserts (Fig. 2).

**Table 4.** Two-way ANOVA of response of seed germination and seedling emergence of *Haloxylon ammodendron* from different sources to rain frequency.

Source	Seed germination				Seedling emergence			
	SS	MS	*F*-value	*P*-value	SS	MS	*F*-value	*P*-value
Rain frequency	0.607	0.202	11.749	<0.001	0.905	0.302	14.168	<0.001
Seed source	1.690	0.563	32.689	<0.001	1.907	0.636	29.862	<0.001
Rain frequency × Seed source	0.493	0.055	3.179	0.004	0.677	0.075	3.532	0.002

### Effects of constant temperature and alternating temperature on seed germination

**Figure 3.**
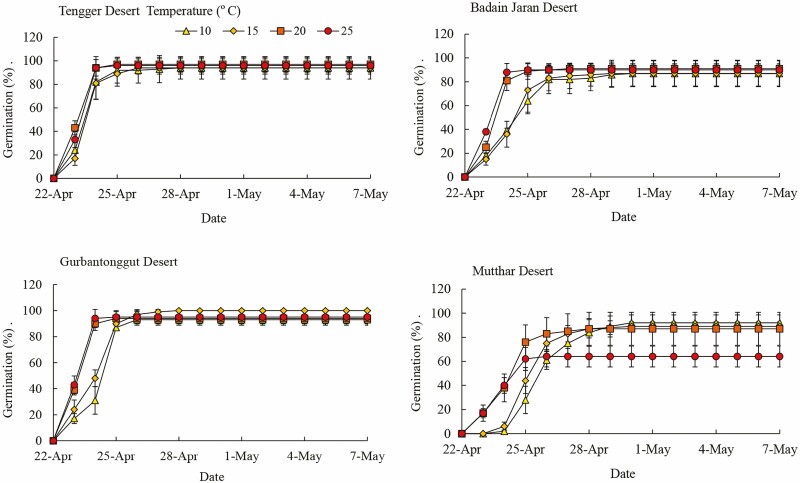
Cumulative daily seed germination of *Haloxylon ammodendron* from four deserts at constant temperatures.

From the Tengger Desert, Badain Jaran Desert and Gurbantonggut Desert, seeds of *H. ammodendron* germinated on the second day at four constant temperatures (Fig. 3). However, seed germination of *H. ammodendron* from the Mutthar Desert was delayed with the decreasing constant temperature, which started on the second day at 20 °C and 25 °C but on the third day at lower constant temperatures.

**Figure 4.**
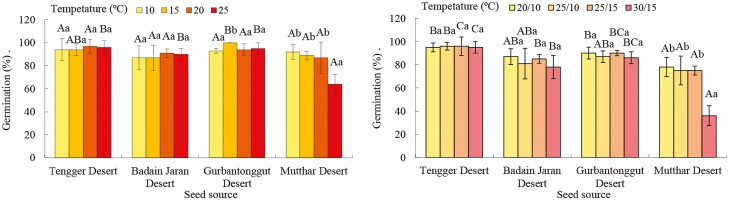
Germination of *Haloxylon ammodendron* seeds from four deserts at constant and alternating temperatures. Different upper-case letters indicate significant differences between seed sources and different lower-case letters indicate significant differences between temperatures.

Seed germination percentage was affected significantly by seed source (*P* < 0.001) and its interactions with constant temperature (*P* < 0.05, Table 5). For *H. ammodendron* from the Tengger Desert, Badain Jaran Desert and Gurbantonggut Desert, seed germination was higher than 80 % at the four temperatures; however, seed germination of *H. ammodendron* seeds from the Mutthar Desert was significantly lower (75 %) at 25 °C (Figs 3 and 4). At the lower temperatures (10 °C to 20 °C), germination percentage was similar for seeds from the four deserts. However, seed germination percentage was significantly higher for Tengger Desert, Badain Jaran Desert and Gurbantonggut Desert seeds than those from the Mutthar Desert at 25 °C (*P* < 0.001) (Fig. 4).

**Table 5.** Two-way ANOVA of response of germination of *Haloxylon ammodendron* seeds from different seed sources to constant and alternating temperatures.

Source	Constant temperature				Alternating temperature			
	SS	MS	*F*-value	*P*-value	SS	MS	*F*-value	*P*-value
Temperature	0.107	0.036	1.528	0.219	0.194	0.065	12.182	<0.001
Seed source	0.628	0.209	8.986	<0.001	0.756	0.252	47.579	<0.001
Temperature × Seed source	0.462	0.051	2.202	0.038	0.314	0.035	6.577	<0.001

**Figure 5.**
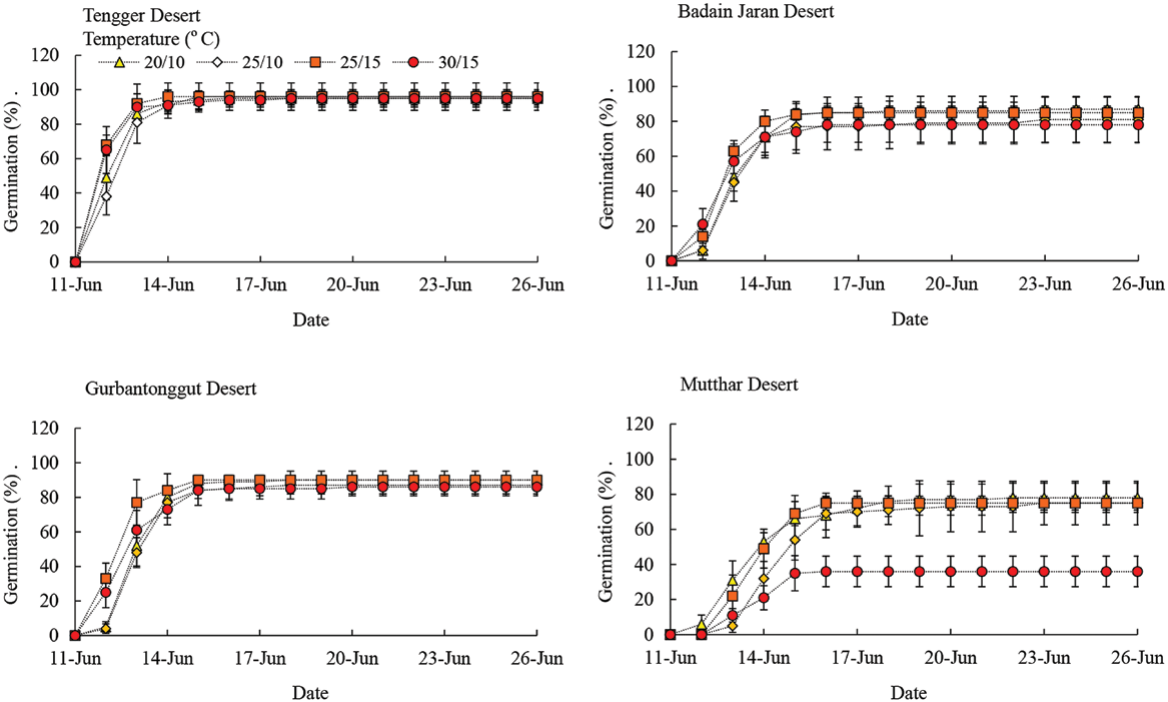
Cumulative daily seed germination of *Haloxylon ammodendron* seeds from four deserts at alternating temperatures.

From the Tengger Desert, Badain Jaran Desert and Gurbantonggut Desert, seeds of *H. ammodendron* germinated on the second day at four alternating temperatures (Fig. 5). However, seed germination of *H. ammodendron* from the Mutthar Desert was delayed with the increasing alternating temperature, which started on the second day at 20/10 °C but on the third day at higher alternating temperatures.

Seed germination percentage of *H. ammodendron* was affected significantly by alternating temperature, seed source and their interactions (*P* < 0.001, Table 5). For *H. ammodendron* seeds from the Tengger Desert, Badain Jaran Desert and Gurbantonggut Desert, maximum germination was 95 %, but only 78 % for seeds from the Mutthar Desert. However, seed germination of *H. ammodendron* from the Mutthar Desert was significantly higher (75 %) at 20/10 °C, 25/15 °C and 25/10 °C than that at 30/15 °C (36 %) (*P* < 0.001, Figs 4 and 5). At 20/10 °C, seed germination percentage was significantly higher in Tengger Desert and Gurbantonggut Desert than that in Mutthar Desert (*P* < 0.05). At 25/10 °C, seed germination percentage was significantly higher for Tengger Desert seeds than Mutthar Desert seeds (*P* < 0.05). At 25/15 °C and 30/15 °C, seed germination percentage was significantly higher from Tengger Desert, Badain Jaran Desert and Gurbantonggut Desert seeds than for Mutthar Desert seeds (*P* < 0.001) (Fig. 4).

## Discussion

The suitable environmental conditions for seed germination are highly unpredictable over space and time in desert ecosystems (Wang *et al.* 2018b). Seeds of most desert plants generally germinate after enough rainfall in the growing season, and have an opportunistic strategy to adapt to the harsh environment (Gutterman 2002). Our experiment results showed that seeds of *H. ammodendron* from four deserts germinated rapidly and well at the rain frequency of 10 times per month totalling 50 mm. Moreover, seed germinated well in a relatively wide range of temperatures varying from 10 °C to 25 °C and from 20/10 °C to 30/15 °C, except the Mutthar Desert seeds that were inhibited by the highest temperature.

### Response of seed germination and seedling emergence to rain frequency change

Seed germination and seedling emergence of *H. ammodendron* from the four deserts were higher at the highest rain frequency (10 times per month) and inhibited by a decrease in rain frequency (Fig. 1). Therefore, the best time for seed germination and seedling emergence of this shrub is late spring or early summer after a few days of rainfall events. The responses of *H. ammodendron* seeds to rain frequency may reflect an opportunistic strategy, which would be an adaptation of the species to desert ecosystems, which is typical in many desert plants. For example, after initial irrigation of 8 mm or 16 mm, subsequent irrigation of 3 mm at 1-day or 2-day intervals resulted in high seedling emergence of three annuals (*Agriophyllum squarrosum*, *Bassia dasyphylla* and *Aristida asdcensionis*) in Mu Us Sandy Land, while irrigation at 4-day or 6-day intervals only favoured seedling emergence of *A. adscensionis* (Tobe *et al.* 2005b). Seedling emergence of three *Artemisia* species was maximal when they were initially and subsequently treated with 16 mm and 3 mm irrigation at 1-day intervals. However, when they were initially and subsequently treated with 8 mm and 3 mm irrigation at 2-day intervals, seedling emergence was almost completely suppressed due to water deficiency in sand (Tobe *et al.* 2006). Similarly, seedling emergence of *Leymus secalinus* is adapted to 150 mm of monthly rain with the frequency of 10–30 times per month, and decreased as rain frequency decreased in Mu Us Sandy Land (Zhu *et al.* 2014). More rains in late spring also enhanced seedling emergence of *Aspidosperma quebracho-blanco*, and the regular rainfall distribution rather than rainfall amount was the most significant factor in the survival of this species in central Argentina (Barchuk *et al.* 2005). Rain frequency was as important as rain amount to seedling emergence of *R. soongarica* since the highest emergence was obtained with a 30 % increase in rain amount and a 50 % reduction in rain frequency at the southern edge of the Badain Jaran Desert (Shan *et al.* 2018). Therefore, rain frequency plays an important role in seed germination and seedling emergence of desert plants. Less frequent rainfall will restrict seed germination and seedling emergence of desert plants in the future.

In the Tengger Desert and Badain Jaran Desert, mean rain frequency is about 3 times per month during the germination season. In the Gurbantonggut Desert and Mutthar Desert, mean rain frequency is about 4 times per month during the germination season (Table 2). Under future climate change, less frequent and larger precipitation events will increase (IPCC 2013); thus, the rain regime in the arid zone would be more unpredictable. Less frequent rain events will inhibit seed germination and seedling emergence of *H. ammodendron*. Moreover, the regeneration of the *H. ammodendron* community might be more sensitive to rain change in the Tengger Desert and Badain Jaran Desert than in the Gurbantonggut Desert and Mutthar Desert, since there is less rain in the two former deserts (about 110 mm) than in the latter desert (about 150 mm) (Table 1).

### Response of seed germination to warming

Seed germination of *H. ammodenron* is adapted to a relatively wide range of temperatures and germination was higher at both constant (10 °C to 25 °C) and alternating temperatures (20/10 °C, 25/10 °C, 25/15 °C and 30/15 °C) for Tengger Desert, Badain Jaran Desert and Gurbantonggut Desert seeds than for Mutthar Desert seeds. Similarly, seeds of *C. korshinskii* from the Ordos Plateau germinated well from 5/15 °C to 25/35 °C (Lai *et al.* 2015). However, germination of *H. ammodendron* seeds from the Mutthar Desert was inhibited by high temperatures (25 °C or 30/15 °C) (Figs 3–5). Based on field observations, seed germination of *H. ammodendron* generally occurs in spring. The inhibition of seed germination in this area may result also from higher air temperatures in the later germination season, which were 26.82 °C and 32.44 °C in May and June, respectively (Table 3). Mean temperature for seed germination was 17.8 ± 0.6 °C in cold deserts (Baskin and Baskin 2014), which was similar to spring air temperatures. High temperature in summer inhibited seed germination of many desert plants. For example, seeds of *Agropyron cristatum*, *A. halodendron*, *C. korshinskii* and *Melilotus suaveolens* from Horqin Sandy Land germinated well at 25/15 °C and 30/20 °C but poorly at 35/25 °C (Lai *et al.* 2016). Warming also inhibited seed germination or seedling emergence of four woody *Banksia* species in South Western Australia (Cochrane *et al.* 2015). Therefore, desert plants may germinate earlier to adapt to global warming in the future.

In deserts of Northwest China, the best time for seed germination of *H. ammodendron* is late spring and early summer, when temperature would be appropriate and sand would be moist after rainfall. Populations of *H. ammodendron* with larger seed mass may have an advantage in desert ecosystems, e.g. Tengger Desert and Gurbantonggut Desert. The effect of seed mass on seed germination and seedling emergence of *H. ammodendron* will need more research, especially under sand burial (Baskin and Baskin 2014). It is predicted that seed germination of *H. ammodendron* will occur earlier under global warming. However, considering both warming and less frequent rainfall, seed germination might be more difficult for *H. ammodendron*, especially in the Mutthar Desert. Therefore, the regeneration of the *H. ammodendron* community should be enhanced in the future by irrigation and seedling transplant.

## Conclusions

In the temperate desert ecosystems, *H. ammodendron* has an opportunistic seed germination strategy which is an adaptation to the unpredictable desert environment. Seeds of *H. ammodendron* from four deserts of Northwest China germinated at high rain frequencies and over a wide range of air temperatures. However, seed germination of *H. ammodendron* was inhibited by the highest temperature of the Mutthar Desert. Therefore, the optimal germination time for *H. ammodendron* is in spring and early summer after rainfall. Under future climate change, seed germination and seedling emergence of *H. ammodendron* would be restricted by warming and decreased frequency of rainfall in the desert ecosystem. The regeneration of *H. ammodendron* community should be promoted by irrigation and seedling transplant.

## Supporting Information

The following additional information is available in the online version of this article. Experiment data of this research was presented in Supporting data 1 and Supporting data 2.

plac048_suppl_Supplementary_Data_S1

plac048_suppl_Supplementary_Data_S2

## Data Availability

The data collected in this study are available as Supporting Information.

## Notes

**Populations & Communities. Chief Editor**: Jean Burns
